# The use of mental health services by Australian adolescents with mental disorders and suicidality: Findings from a nationwide cross-sectional survey

**DOI:** 10.1371/journal.pone.0231180

**Published:** 2020-04-10

**Authors:** Md. Irteja Islam, Rasheda Khanam, Enamul Kabir

**Affiliations:** 1 Maternal and Child Health Division, International Centre for Diarrhoeal Disease Research, Bangladesh (icddr,b), Dhaka, Bangladesh; 2 Centre for Health, Informatics, and Economic Research and School of Commerce, University of Southern Queensland, Queensland, Australia; BRAC, BANGLADESH

## Abstract

**Objectives:**

Mental disorders and suicidality among adolescents have been identified as a major public health concern worldwide; however, they often do not get the necessary attention from parents, school and health professional, and therefore are left untreated. This study aimed to investigate the factors associated with the use of mental health services among Australian adolescents aged 13–17 with mental disorders and/or suicidality.

**Methods:**

Adolescents aged 13–17 (n = 2134) from Young Minds Matter (YMM): the Second Australian Child and Adolescent Survey of Mental Health and Wellbeing were included in this study. The YMM is a cross-sectional nationwide survey, in which information was collected from both parents and adolescents (aged 13–17 years). Both bivariate and multivariate analyses were conducted to identify the factors that have an impact on the use of mental health services (outcome variable) in two subsamples: (1) adolescents with mental disorder and (2) adolescents with suicidality.

**Results:**

Overall, 740 (34.7%) and 168 (7.9%) adolescents reported a mental disorder and/or suicidality, respectively. The incidence of seeking any service was higher among adolescents with suicidality (approximately 50%) compared to those with a mental disorder (about 30%). Girls, older age-group (15–17), adolescents living with disadvantaged families (lower-income, less educated and unemployed parents), those who had multiple mental disorders and history of substance use were most likely to use mental health services regardless of mental disorder and suicidality. Health services and online services were the most popular type of mental health service among adolescents aged 13–17 across two subgroups, while, school and telephone services were less utilized.

**Conclusions:**

Many adolescents with mental disorders and/or suicidality do not use mental health services. The findings indicate differences in factors associated with the use of mental health services among adolescents with mental disorder and suicidality. Further research is needed to address the specific barriers that limit the use of the services.

## Introduction

Mental disorders and suicidality (ideation, plan or attempt) among adolescents is a major public health concern due to its high prevalence and the significant burden it places on society [1–4]. Not only are mental disorders and suicidality frequently correlated, but both are significant contributors to premature mortality and disabling conditions and are expensive worldwide [1–3, 5]. Globally, suicide is considered as one of the topmost causes of death among 10–24 year-olds [6, 7]. For example, recent studies in the United States suggest that the suicide rate among this age-group climbed 56% in 2017 from 2007 to rank it as the second leading cause of death [8]. It is also recognized as the leading cause of death among Australian aged 15–24 years [6, 9]; the Australian Bureau of Statistics recently reported that the age-specific suicide-rate in individuals aged 15–19 and 20–24 were respectively 12.3 and 14.5 deaths per 100,000 in 2018, which is higher compared to the rate reported in 2017 [10]. Further, the presence of a mental disorder is found to be significantly associated with suicidality with almost 20% of Australian adolescents aged 12–17 years who attempted suicide have a diagnosable mental disorder [11]. According to the most recent Australian mental health survey, one in 7 children and adolescents aged 4–17 years have at least one mental disorder [12], with the highest percentage of attention deficit hyperactivity disorder (ADHD), which is about 7.4%, followed by anxiety disorders (6.9%), major depressive disorder (2.8%) and conduct disorder (2.1%) [3, 12]. However, mental disorders and suicidality among children and adolescents are often unidentified by their parents and school teachers and left untreated by any health professionals [4, 13, 14].

In Australia, only 65% of adolescents aged 12–17 years with a mental disorder and/or suicidality have used mental health services [3, 15, 16]. With so many adolescents with mental disorder and/or suicidality not receiving mental health services [13, 17], there is an urgent need to investigate the factors associated with service utilization involving this group of people in particular.

Previous studies have found several factors that are associated with the service use [1, 3, 4, 18, 19]. For example, Cuffle et al. [18] indicated that gender may have significant impact on mental health service uses, while, other studies showed that gender may not be associated with service use when respondents were aged under-19 in particular [4, 18, 20]. Age is another important factor of mental health service use [3], with multiple studies claiming that children aged more than 11-years have a higher probability of mental health problems and potential of using mental health services [3, 21]. Previous studies also examined the impact of household income, education and occupation of parents’ on service use for mental health problems [3, 4, 13, 14]. Steele et al. [22] showed that adolescents from low-income families were less likely to access services, while, few other studies found no relationship between household income and service use in adolescents [3, 23]. Similarly, several studies also showed that lower parental education was associated with lower use of mental health services [3, 24], while other studies [25] did not find such association between parental education and service use due to mental health problems. Previous research also suggested that other family-related factors such as family type, family functioning and family stress may influence the use of mental health services [4, 26]. For instance, findings from a study in Australia showed that adolescents from blended-families were less likely to access mental health services compared to those from single-parent families [26]. Finally, co-morbid illness factors such as co-occuring mental disorders and suicidality in an individual are also found to be strong predictors of using mental health services among adolescents due to the fact that mental disorders and suicidality are closely interrelated [3, 13, 26]. For example, Brent et al. [27] reported that approximately 35–50% of adolescents with mental disorders attempted suicide. In Australia, these differences may be even stronger with the fact that policymakers, families and health care providers have struggled to understand how this critical public health problem can be handled [3, 13, 26].

Several studies have examined the factors associated with mental health service use among adolescents across countries [1, 14, 18, 24, 28], However, most of the studies have either investigated children as a group (up to age less than 18), adolescents with youth (age 10–24), or only adults (age more than 18). Similarly, Johnson et al. [26] and Vu et al. [3] examined the factors related to mental health service use among children aged 4–17 years in Australia. However, the authors of the studies [3, 26] did not examine the impact of mental disorder and/or suicidality in an adolescent on service use. Moreover, Johnson et al. [26] only investigated the differences in factors of only two types of mental health services (i.e. health and school service) and omitted telephone and online service, which are thought to be the recent preferred ones among adolescent. To our knowledge, no individual study in Australia has directly compared the differences in factors associated with service use among adolescents aged 13–17 years with mental disorders and/or suicidality. Therefore, this study aimed to examine the impact of different factors on mental health services use in adolescents aged 13–17 years, who were diagnosed with mental disorders and/or reported suicidality.

## Methodology

### Data source

Cross-sectional data were analyzed from Young Minds Matter (YMM): the Second Australian Child and Adolescent Survey of Mental Health and Wellbeing, which was conducted during 2013–2014 by the University of Western Australia (UWA) thorough the Telethon Kids Institute in collaboration with Roy Morgan Research and the Australian Government Department of Health [17, 29] Since the YMM was ethically approved by the Human Research Ethics Committee of the Australian Government Department of Health and by the Human Research Ethics Committee of the UWA [26, 29], ethical approval was not required for this paper as we only used data from the YMM survey.

In brief, YMM employed a multi-stage, area-based random sampling technique and designed to be representative of households with children and adolescents aged 4–17 years in Australia [15, 29]. If more than one qualifying child was present in the household, the sample randomly included a single child [29, 30]. In total, 6310 parents of children and adolescents aged 4–17 years (55% eligible households) participated voluntarily in the survey through a face-to-face interview using a structured questionnaire, and 2967 adolescents aged 11–17 years (89% eligible youth) completed computer-based self-reported questionnaires privately to provide information on risk behaviours (e.g. suicidality, self-harm, substance use, bullying) and service use [17, 29]. However, the survey excludes the most remote areas, homeless adolescents, adolescents living in any institutional care and in households where the interviews could not be conducted in English. A more detailed description of the methodology used for the survey will be found elsewhere [29].

### Measures

#### Services

Different types of mental health services used by adolescents aged 13-17years were considered as an outcome variable in this study. All consenting parents were asked a series of questions regarding services used for emotional or behavioural problems in the past 12-months. Self-reported information of service use was restricted to adolescents aged 13–17 years to capture true important findings as adolescents’ transitioned through puberty due to the perceived sensitive nature of the questions. The mental health services accessed by the adolescents included (I) health services, (II) school services, (III) telephone counselling services and (IV) online self-help services (only available in child-data). Health services included services provided by general medical practitioners including family physicians and paediatricians, psychiatrists, psychologists, counsellors, psychotherapists, mental health nurses and social workers in a mental health speciality setting or any setting such as hospital inpatient, outpatient and emergency department. Examples of school services included any school or educational institution-based programs that consisted of any special schools, special classes within a school, and school-based therapies that a child was attending [26]. Each service was a binary variable and responses were coded as ‘1’ for Yes and ‘0’ for No. Lastly, for both parent-data and child-data, all types of services were combined to create an additional variable ‘Any service’ in our analysis: whether the adolescent had ever accessed any of the four services for a mental disorder and/or a behavioural problem like suicidality. Responses included ‘Yes’ (coded as 1 if using at least one of these services) or ‘No’ (coded as 0 otherwise).

#### Mental disorders

Mental disorders in the 12-months preceding the survey among adolescents were assessed by the Diagnostic Interview Schedule for Children–Version IV (DISC-IV) [17], which implements the criteria for mental disorders set out in the Diagnostic and Statistical Manual of Mental Disorders–Version IV, formed by the American-Psychiatric-Association [31]. DISC-IV modules were completed by parents as well as by adolescents. Mental disorders included major depressive disorder, attention deficit hyperactivity disorder (ADHD), conduct disorder, and four types of anxiety disorders—social phobia, separation anxiety disorder, generalized anxiety disorder, and obsessive-compulsive disorder. In this paper, binary variables were created to identify the presence of any mental disorders using only parent-data, as it only provides information on the diagnosis of each type of mental disorder among children and adolescents. Initially, social phobia, separation anxiety disorder, generalized anxiety disorder and obsessive-compulsive disorder were categorized into one category—anxiety disorders. We then added a variable ‘mental disorder’ in our analysis: whether the adolescent has had any of the following types of mental disorder—anxiety disorders, major depressive disorder, ADHD and conduct disorder. Responses included ‘Yes’ (coded as 1 if a child experiences at least one of these disorders) or ‘No’ (coded as 0 if otherwise). In addition, a variable ‘number of mental disorder’ was included with two categories (single/multiple) in the analysis.

#### Suicidality

Items measuring suicidality (suicidal ideation, plans and attempts) were collected from the Standard High School questionnaires of the CDC 2009 YRBS [32]. However, due to perceived sensitive nature of the questions, suicidality was measured only in adolescents aged 12–17 years. All yes-no response options were coded 1 for ‘Yes’ and 0 for ‘No’. Suicidal ideation was measured by the following question, *‘During the past 12 months*, *did you ever seriously consider attempting suicide*?*’* For ideators, *suicide plans and attempts* were assessed with two questions, respectively; *‘During the past 12 months*, *did you make a plan about how you would attempt suicide*?*’; ‘Did you attempt suicide during the past 12 months*?*’* Responses to these questions were used to classify ideators into two groups: (I) suicidal ideation without plan and attempt; (II) suicidal ideation with a plan and/or attempt. Note that information regarding suicidality captured from only child-data, where confidentiality was maintained regarding all responses and not shared with consenting parents [11].

#### Sociodemographic factors

The source of information for all the sociodemographic factors was parent data except for substance use by the child which was taken from child-data. Covariates included—adolescents’ age (13-≤15 vs. 15–17 years), gender (boys vs. girls), remoteness (cities vs. regional/remote), household income/year (more than $130000 as high, $52000-$129999 as medium and less than $52000 as low), parental education (bachelor, diploma and year-10/11), parental employment (employed vs. unemployed), family type (adolescents from original parents vs. adolescents from other families such as step and blended), family functioning (very good/good vs. fair/poor), The index of relative socio-economic advantage and disadvantage (IRSAD) quintile (lowest, second, third, fourth and highest), substance use by the child (yes vs. no).

### Data analysis

**Fig 1:**
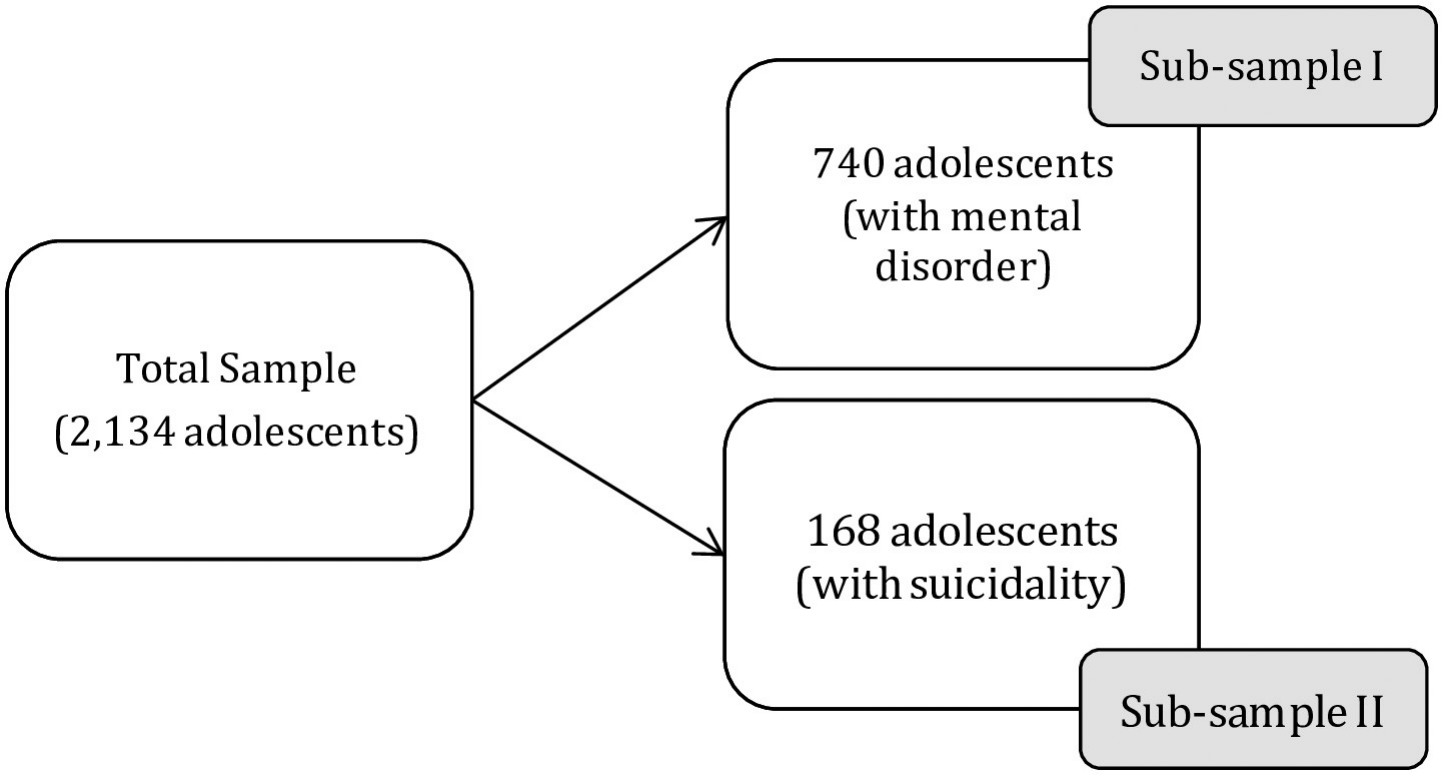
Flow-chart of selecting sample and sub-sample population for the analysis.

The analysis in this paper uses the whole sample and several sub-samples as follows (Fig 1):

- When assessing service use the sample is restricted to adolescents aged 13–17 years across two datasets (self-reported child-data and parent-data) to maintain age comparability across the survey, the whole sample was used (*n* = 2134).
- When investigating service use by adolescents with mental disorders identified from parent-data on the DISC-IV, the analysis involved adolescents aged 13–17 years who had a mental disorder (n = 740).
- When examining service use by adolescents with suicidality, the analysis was undertaken on the adolescents aged 13–17 years who reported suicidality exclusively in the self-reported child-data (n = 168).
- The ‘Don’t know’ responses were omitted

Initially, descriptive statistics on sociodemographic and risk-behaviour correlates were calculated and stratified by mental disorder and suicidality status among adolescents aged 13–17 years. Chi-square tests of significance were used to describe and compare the sample characteristics of adolescents with mental disorder and suicidality. Bivariate associations also measured between the variables and their distributions over the outcome variables (various services used by the adolescents) among the adolescents with mental disorder and suicidality, separately. All associations yielding a P-value<0.05 were used to build a binary logistic regression model. Factors related to service use in adolescents with mental disorders and suicidality was assessed with regression methods using the Stata/SE 14.1. All the estimates were weighted to represent 13-17-year-olds in the Australian population, in which weights were calculated according to the Deville and Sarndal’s generalized raking procedure [33]. The strength of the associations between the use of various mental health services and sociodemographic factors was estimated employing odds ratios (OR) and 95% confidence intervals.

## Results

Table 1 provides the distribution of socio-demographic data and risk behaviour correlates for the whole sample and among adolescents with mental disorders and suicidality, separately. Of the 2134 adolescents included in the analyses, the mean age was 15.4, 52.1% were girls, and adolescents from cities were oversampled. More than 40% of adolescents were from other (step, blended and others) than original family and 42.1% were reported of using a substance. Of the total sample, 34.4% of adolescents had a mental disorder and 7.9% had experienced suicidal ideation. Adolescents who had a mental disorder were more likely from major cities (66.8%) and the low-medium-income household (74.3%) compared to a high-income household (25.7%). Girls were twice as likely (72.0% vs. 28%, *p*<0.001) to report suicidality as boys. Adolescents who reported suicidality were more likely to have a history of using any substances (79.2%, *p*<0.001).

**Table 1:** Sample characteristics (i.e. Adolescents aged 13–17 years).

Characteristics	Total	Mental Disorder^§^		Suicidality^¥^	
	n (%)	n (%)	*p*- value	n (%)	*p*- value
Total	2134 (100.0)	740 (34.7)		168 (7.9)	
Age (Mean = 15.4, SD = 1.39)					
13 to ≤15	891 (41.8)	328 (44.3)	0.079	51 (30.4)	0.002
>15 to 17	1243 (58.2)	412 (55.7)		117 (69.6)	
Gender					
Boys	1112 (52.1)	390 (52.7)	0.689	47 (28.0)	<0.001
Girls	1022 (47.9)	350 (47.3)		121 (72.0)	
Remoteness					
Cities	1372 (64.3)	494 (66.8)	0.083	102 (60.7)	0.211
Regional/Remote	762 (36.7)	246 (33.2)		66 (39.3)	
Household income^					
Low	493 (23.1)	208 (28.1)	<0.001	49 (29.2)	0.015
Medium	983 (46.1)	342 (46.2)		82 (48.8)	
High	658 (30.8)	190 (25.7)		37 (22.0)	
Parents’ educational level					
Bachelor	684 (32.1)	198 (26.8)	0.001	46 (27.4)	0.362
Diploma	771 (36.1)	285 (38.5)		65 (38.7)	
Year 10/11	679 (31.8)	257 (34.7)		57 (33.9)	
Parents’ employment status					
Employed	1631 (76.4)	531 (71.8)	<0.001	118 (70.2)	0.023
Unemployed	503 (23.6)	209 (28.2)		50 (29.8)	
Family type^j^					
Original	1275 (59.7)	404 (54.6)	<0.001	86 (51.2)	0.011
Other	859 (40.3)	336 (45.4)		82 (48.8)	
Family functioning					
Very good/Good	1758 (82.4)	564 (76.2)	<0.001	122 (72.6)	<0.001
Fair/Poor	376 (17.6)	176 (23.8)		46 (27.4)	
IRSAD quintile^e^					
Lowest	323 (15.1)	135 (18.2)	0.001	38 (22.6)	0.036
Second	370 (17.3)	147 (19.9)		28 (16.7)	
Third	454 (21.3)	157 (21.2)		38 (22.6)	
Fourth	474 (22.2)	151 (20.4)		28 (16.7)	
Highest	513 (24.0)	150 (20.3)		36 (21.4)	
Substance use by the child†					
No	1225 (57.4)	403 (54.5)	0.045	35 (20.8)	<0.001
Yes	909 (42.6)	337 (45.5)		133 (79.2)	

Data are shown as n (%)

P-value of association with different mental health services

^§^No. of children having any of the following mental disorders—ADHD or Major depressive disorder or Anxiety disorder (General anxiety/Separation anxiety/Obsessive Compulsive Disorder/Social phobia) or conduct disorder

^¥^No. of children seriously considered attempting suicide in the past 12 months

^Household income: Low (<$52000), Medium ($52000-$129999) and High (>$130000)

^j^Family type: original families means children are natural, adopted, or foster child of both parents, and no step child; other families include step, blended and children from families who are not natural, adopted, foster or step of either parent

^e^IRSAD: “The Index of Relative Socio-economic Advantage and Disadvantage (IRSAD): Summarizes information about the economic and social conditions of people and households within an area, including both relative advantage and disadvantage measures. Low indicates relatively greater disadvantage and a lack of advantage in general and high score indicates a relative lack of disadvantage and greater advantage in general.”

^†^Ever seriously try cigarette smoking, drink alcohol, cannabis or any other illegal drugs

**Fig 2:**
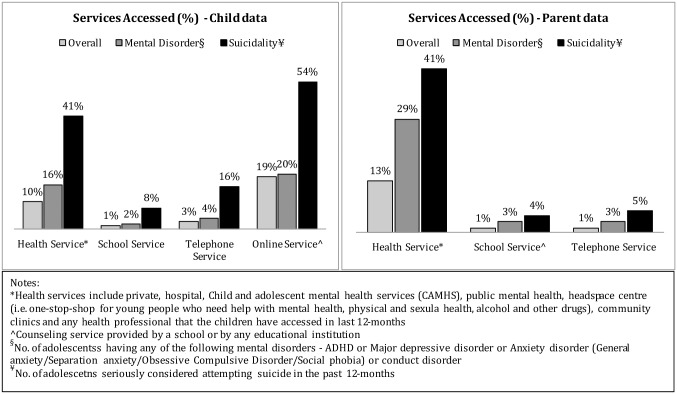
Distribution of mental health services. *Health service include private, hospital, Child and adolescent mental health service (CAMHS), public mental health.

Fig 2 depicts the distribution of mental health services in the whole sample and two sub-samples (adolescents with mental disorders and adolescents with suicidality). According to the child data, online service was the preferred one in each group; followed by health services. While, parent data shows that health services were most likely to be accessed by the adolescents. Fig 2 also shows that school services were the least used service by adolescent with mental disorder and suicidality in both child data and parent data. In addition, it shows though the percentages is low compared to other services, however, the number of adolescents who used telephone counselling services was higher, as shown by child data compared to parent data.

### Adolescents with mental disorders: Sociodemographic factors vs. mental health services

Table 2 demonstrates in both child and parent data that a greater proportion of older age-group (>15–17 years) tended to access all or at least one of the mental health services including online services compared to the younger group aged 13–15 years. Both data also reported that more girls used all types of mental health services than boys. Adolescents from low-medium income households utilized more health services, compared to adolescents from high-income households which may be due to that adolescents from low-medium income households are more likely to have a mental disorder. Educational background of the parents did not have such differentiated impact on the utilization of any services. Parents’ employment found to have good impact on mental health service use among adolescents given that employed parents are more likely to be educated so they have better health information and more aware of the consequence of mental disorders and suicidality. Table 2 also shows that remoteness, family functioning and IRSAD quintile did not have a significant impact on most of the mental health services; except for health services in child data where IRSAD quintile found to have a significant impact. As expected, adolescents who had a history of substance use accessed more mental health services compared to others, except for school services in parent data. Also, adolescents with multiple mental disorders were found to have a significant impact on the service use compared to those with a mental disorder, except for telephone services in child data.

**Table 2:** Bivariate analysis between mental health services and sociodemographic factors in adolescents with mental disorders (n = 740).

Characteristics	Child data					Parent data			
	Health Service	School Service	Telephone Service	Online Service	Any Service	Health Service	School Service	Telephone Service	Any Service
Total	120 (16.2)	13 (1.8)	29 (3.9)	148 (20.0)	203 (27.4)	211 (28.5)	19 (2.6)	19 (2.6)	221 (29.9)
Age									
13 to £15	37 (30.8)	5 (38.5)	12 (41.4)	51 (34.5)	74 (36.4)	75 (35.5)	12 (63.2)	3 (15.8)	79 (35.7)
>15 to 17	83 (69.2)	8 (61.5)	17 (58.6)	97 (65.5)	129 (63.5)	136 (64.5)	7 (36.8)	16 (84.2)	142 (64.3)
*p*- value	0.001	0.668	0.745	0.007	0.008	0.002	0.094	0.011	0.002
Gender									
Boys	42 (35.0)	3 (23.1)	13 (44.8)	47 (31.8)	79 (38.9)	97 (46.0)	9 (47.4)	4 (21.0)	101 (45.7)
Girls	78 (65.0)	10 (76.9)	16 (55.2)	101 (68.2)	124 (69.0)	114 (54.0)	10 (52.6)	15 (79.0)	120 (54.3)
*p*- value	<0.001	0.031	0.386	<0.001	<0.001	0.021	0.637	0.005	0.013
Remoteness									
Cities	84 (70.0)	8 (61.5)	16 (55.2)	105 (71.0)	144 (70.9)	130 (61.6)	11 (57.9)	15 (78.9)	138 (62.4)
Regional/Remote	36 (30.0)	5 (38.5)	13 (44.8)	43 (29.0)	59 (29.1)	81 (38.4)	8 (42.1)	4 (21.1)	83 (37.6)
*p*- value	0.410	0.687	0.177	0.226	0.138	0.061	0.406	0.253	0.104
Household income									
Low	40 (33.3)	3 (23.1)	11 (37.9)	40 (27.0)	61 (30.0)	74 (35.1)	6 (31.6)	5 (26.3)	78 (35.3)
Medium	55 (45.9)	8 (61.5)	13 (44.8)	72 (48.7)	94 (46.3)	92 (43.6)	9 (47.4)	10 (52.6)	98 (44.3)
High	25 (20.8)	2 (15.4)	5 (17.3)	36 (24.3)	48 (23.7)	45 (21.3)	4 (21.0)	4 (21.1)	45 (20.3)
*p*- value	0.259	0.512	0.390	0.800	0.662	0.021	0.882	0.836	0.009
Parents’ education									
Bachelor	32 (26.6)	4 (30.8)	4 (13.8)	41 (27.7)	57 (28.1)	55 (26.1)	8 (42.1)	4 (21.1)	58 (26.2)
Diploma	50 (41.7)	6 (46.1)	16 (55.2)	63 (42.6)	83 (40.9)	79 (37.4)	7 (36.8)	10 (52.6)	84 (38.0)
Year 10/11	38 (31.7)	3 (23.1)	9 (31.0)	44 (29.7)	63 (31.0)	77 (36.5)	4 (21.1)	5 (26.3)	79 (35.8)
*p*- value	0.685	0.671	0.123	0.336	0.429	0.817	0.249	0.440	0.930
Parents’ employment									
Employed	74 (61.7)	10 (76.9)	20 (69.0)	103 (69.6)	138 (68.0)	137 (64.9)	14 (73.7)	13 (68.4)	144 (65.2)
Unemployed	46 (38.3)	3 (23.1)	9 (31.0)	45 (30.4)	65 (32.0)	74 (35.1)	5 (26.3)	6 (31.6)	77 (34.8)
*p*- value	0.007	0.676	0.733	0.514	0.161	0.009	0.850	0.744	0.009
Family type									
Original	56 (46.7)	5 (38.5)	13 (44.8)	76 (51.4)	103 (50.7)	93 (44.1)	9 (47.4)	7 (36.8)	99 (44.8)
Other	64 (53.3)	8 (61.5)	16 (55.2)	72 (48.6)	100 (49.3)	118 (55.9)	10 (52.6)	12 (63.2)	122 (55.2)
*p*- value	0.057	0.239	0.281	0.376	0.195	<0.001	0.522	0.115	<0.001
Family functioning									
Very good/Good	89 (74.2)	10 (76.9)	24 (82.8)	114 (77.0)	155 (76.4)	155 (73.5)	17 (89.5)	14 (73.7)	162 (73.3)
Fair/Poor	31 (25.8)	3 (23.1)	5 (17.2)	34 (23.0)	48 (23.6)	56 (26.5)	2 (10.5)	5 (26.3)	59 (26.7)
*p*- value	0.565	0.952	0.399	0.796	0.957	0.266	0.169	0.793	0.225
IRSAD quintile									
Lowest	21 (17.5)	3 (23.1)	7 (24.2)	25 (16.9)	34 (16.7)	39 (18.5)	2 (10.5)	4 (21.1)	41 (18.5)
Second	13 (10.9)	1 (7.7)	3 (10.3)	20 (13.5)	28 (13.8)	42 (19.9)	3 (15.8)	1 (5.2)	43 (19.5)
Third	36 (30.0)	4 (30.7)	10 (34.5)	33 (22.3)	51 (25.1)	53 (25.1)	5 (26.3)	7 (36.8)	57 (25.8)
Fourth	25 (20.8)	2 (15.4)	6 (20.7)	37 (25.0)	48 (23.7)	35 (16.6)	4 (21.1)	4 (21.1)	37 (16.7)
Highest	25 (20.8)	3 (23.1)	3 (10.3)	33 (22.3)	42 (20.7)	42 (19.9)	5 (26.3)	3 (15.8)	43 (19.5)
*p*- value	0.025	0.747	0.205	0.176	0.061	0.373	0.845	0.329	0.255
Substance Use by the child									
No	42 (35.0)	3 (23.0)	8 (27.6)	52 (35.1)	79 (38.9)	92 (43.6)	9 (47.4)	6 (31.6)	100 (45.3)
Yes	78 (65.0)	10 (76.9)	21 (72.4)	96 (64.9)	124 (61.1)	119 (56.4)	10 (52.6)	13 (68.4)	121 (54.7)
*p*- value	<0.001	0.022	0.003	<0.001	<0.001	<0.001	0.529	0.042	0.001
No of mental disorders									
Single	58 (48.3)	4 (30.8)	16 (55.2)	90 (60.8)	121 (59.6)	101 (47.9)	6 (31.6)	5 (26.3)	107 (48.4)
Multiple	62 (51.7)	9 (69.2)	13 (44.8)	58 (39.2)	82 (40.4)	110 (52.1)	13 (68.4)	14 (73.7)	114 (51.6)
*p*- value	<0.001	0.003	0.103	0.017	0.001	<0.001	<0.001	<0.001	<0.001

Data are shown as n (%)

P-value of association with different mental health services

A binary logistic model was used to investigate the factors associated with the use of various mental health services among adolescents with mental disorders (Table 3). According to child data, girls were respectively 2.62 (95% CI: 1.61–4.28), 2.50 (95% CI: 1.62–3.88) and 1.95 (95% CI: 1.31–2.91) times more likely to use health service, online service and any services compared to boys, however, parent data did not show such differences in gender. Child data also shows adolescents of unemployed parents’ were 1.84 times (95% CI: 1.04–3.24) more likely to utilize health services compared to those from employed parents’. In parent data, it is found that adolescents born in middle-income families tended to utilize telephone services 5.54 times more compared to adolescents from low and high-income households and only health services or any services were significantly associated with family type. Both data shows that adolescents who had a history of substance use were significantly associated with all types of services compared to those who do not have any exposure to substances except for school service in parent data. Further, Table 3 shows that adolescents with more than one mental disorder were more likely to use health service, school service or any services compared to those with single disorder, as reported by child data and parent data.

**Table 3:** Factors associated with mental health service uses in sub-sample I (binary regression).

Sociodemographic factors		Child data					Parent data			
		Health Service	School Service	Telephone Service	Online Service	Any Service	Health Service	School Service	Telephone Service	Any Service
		OR (95% CI)	OR (95% CI)	OR (95% CI)	OR (95% CI)	OR (95% CI)	OR (95% CI)	OR (95% CI)	OR (95% CI)	OR (95% CI)
Age (Ref. 13 to ≤15)	>15 to 17	1.42 (0.80, 2.55)	0.42 (0.09, 1.91)	0.65 (0.22, 1.84)	1.14 (0.70, 1.85)	1.15 (0.75, 1.76)	0.99 (0.64, 1.53)	0.18***(0.04, 0.85)	1.25 (0.23, 6.75)	0.99 (0.64, 1.51)
Gender (Ref. Boys)	Girls	2.62*(1.61, 4.28)	3.29 (0.75, 14.45)	1.15 (0.49, 2.69)	2.50*(1.62, 3.88)	1.95**(1.31, 2.91)	1.22 (0.82, 1.80)	1.25 (0.44, 3.51)	3.19 (0.84, 12.06)	1.22 (0.83, 1.80)
Remoteness (Ref. Cities)	Regional/Remote	1.01 (0.56, 1.79)	2.27 (0.60, 8.56)	1.37 (0.54, 3.47)	0.85 (0.51, 1.40)	0.76 (0.48, 1.20)	1.17 (0.73, 1.89)	2.17 (0.51, 9.25)	1.22 (0.33, 4.47)	1.16 (0.72, 1.84)
Household income (Ref. Low)	Medium	0.85 (0.44, 1.62)	2.60 (0.53, 12.63)	0.53 (0.18, 1.51)	1.04 (0.55, 1.93)	0.83 (0.49, 1.41)	0.84 (0.49, 1.45)	0.98 (0.23, 4.11)	5.54***(1.07, 28.60)	0.89 (0.52, 1.52)
	High	0.63 (0.29, 1.33)	1.58 (0.16, 15.43)	0.40 (0.12, 1.32)	0.69 (0.32, 1.45)	0.59 (0.31, 1.13)	0.89 (0.45, 1.77)	0.54 (0.06, 4.44)	2.98 (0.46, 19.30)	0.81 (0.41, 1.58)
Parents’ education (Ref. Bachelor)	Diploma	1.02 (0.56, 1.85)	0.66 (0.16, 2.63)	2.65 (0.78, 9.00)	0.98 (0.58, 1.65)	0.87 (0.52, 1.43)	0.61 (0.36, 1.05)	0.22 (0.05, 1.00)	0.74 (0.19, 2.78)	0.59 (0.35, 1.00)
	Year 10/11	0.58 (0.30, 1.12)	0.30 (0.07, 1.23)	1.01 (0.30, 3.41)	0.66 (0.32, 1.45)	0.59***(0.31, 1.13)	0.66 (0.36, 1.21)	0.23***(0.07, 0.72)	0.54 (0.17, 1.69)	0.61 (0.34, 1.10)
Parents’ employment (Ref. Employed)	Unemployed	1.84***(1.04, 3.24)	0.98 (0.27, 3.51)	0.91 (0.39, 2.14)	1.38 (0.80, 2.40)	1.50 (0.94, 2.39)	1.29 (0.82, 2.03)	2.33 (0.60, 8.97)	3.03 (0.84, 10.86)	1.36 (0.87, 2.13)
Family type (Ref. Original)	Other	1.40 (0.86, 2.27)	2.11 (0.59, 7.47)	1.39 (0.59, 3.27)	0.96 (0.60, 1.53)	1.07 (0.70, 1.64)	2.06**(1.33, 3.19)	0.88 (0.26, 2.95)	4.29 (0.97, 18.99)	1.96**(1.28, 3.01)
Family functioning (Ref. Very good/Good)	Fair/Poor	1.06 (0.61, 1.82)	1.31 (0.35, 4.89)	0.47 (0.16, 1.37)	0.93 (0.57, 1.53)	0.91 (0.58, 1.42)	1.02 (0.64, 1.60)	0.64 (0.13, 3.00)	0.63 (0.14, 2.69)	1.05 (0.67, 1.67)
IRSAD quintiles (Ref. Lowest)	Second	0.35***(0.14, 0.83)	0.11 (0.00, 1.47)	0.53 (0.12, 2.35)	0.60 (0.27, 1.31)	0.60 (0.30, 1.17)	1.14 (0.56, 2.31)	0.39 (0.04, 3.45)	0.35 (0.02, 5.07)	1.17 (0.58, 2.35)
	Third	1.45 (0.64, 3.28)	0.79 (0.11, 5.49)	1.67 (0.46, 6.09)	0.96 (0.43, 2.13)	1.21 (0.62, 2.35)	1.29 (0.66, 2.52)	1.79 (0.41, 7.82)	2.57 (0.46, 14.22)	1.47 (0.75, 2.88)
	Fourth	1.24 (0.53, 2.90)	0.46 (0.08, 2.41)	0.88 (0.25, 3.07)	1.27 (0.60, 2.72)	1.37 (0.70, 2.65)	0.91 (0.45, 1.83)	1.77 (0.34, 9.13)	1.59 (0.29, 8.74)	1.05 (0.53, 2.07)
	Highest	1.10 (0.47, 2.57)	0.73 (0.13, 3.91)	0.62 (0.12, 3.16)	1.03 (0.46, 2.29)	0.94 (0.46, 1.90)	1.30 (0.61, 2.77)	1.91 (0.39, 9.31)	1.95 (0.31, 12.00)	1.34 (0.63, 2.83)
Substance Use (Ref. No)	Yes	1.74***(0.99, 3.05)	5.16***(1.01, 26.28)	4.56** (1.65, 12.56)	2.02**(1.23, 3.33)	1.82**(1.16, 2.87)	2.26**(1.40, 3.64)	2.77 (0.72, 10.63)	8.73***(1.02, 74.17)	2.08**(1.31, 3.23)
No of Mental disorders (Ref. Single)	Multiple	3.23*(1.98, 5.27)	6.46***(1.58, 26.44)	1.18 (0.51, 2.73)	1.37 (0.89, 2.11)	1.78**(1.18, 2.69)	3.34*(2.16, 5.18)	9.96*(3.14, 31.53)	3.90 (0.93, 16.28)	3.35*(2.17, 5.18)

OR = odds ratio; CI = confidence interval

Level of Significance Considered: P<0.05***, P<0.01**, P<0.001*

Survey weight adjusted

### Adolescents with suicidality: Sociodemographic factors vs. mental health services

The bivariate analysis between mental health service use and sociodemographic factors among adolescents with suicidality (child data and parent data, Table 4) illustrates that more girls accessed at least one of the health services than boys and older (>15–17) age-group use services more compared to younger (13-≤15) age-group. Online service, which was limited in child data, shows that76.7% of the adolescents who accessed online services were aged between >15–17 years, more girls (80%) use this service than boys and majority were from low-medium income families. Child data also shows that telephone service use is higher among adolescents from step/blended families compared to those living with their original parents. Parent data shows that adolescents with poor family functioning were less likely to utilize any services compared to those with good family functioning. As expected, both data shows among suicidal adolescents who reported of substance use accessed at least one service compared to those who did not use substances, and adolescents with a mental disorder used health services than by those without a mental disorder.

**Table 4:** Bivariate analysis between mental health services and sociodemographic factors in adolescents with suicidality (n = 168).

Characteristics	Child data					Parent data			
	Health Service	School Service	Telephone Service	Online Service	Any Service	Health Service	School Service	Telephone Service	Any Service
Total	69 (41.1)	13 (7.7)	26 (15.5)	90 (53.6)	110 (65.5)	69 (41.1)	7 (4.2)	9 (5.4)	72 (42.9)
Age									
13 to ≤15	19 (27.5)	6 (46.2)	8 (30.8)	21 (23.3)	27 (24.5)	16 (23.2)	5 (71.4)	1 (11.1)	18 (25.0)
>15 to 17	50 (72.5)	7 (53.8)	18 (69.2)	69 (76.7)	83 (75.5)	53 (76.8)	2 (28.6)	8 (88.9)	54 (75.0)
*p*- value	0.507	0.197	0.096	0.033	0.024	0.092	0.016	0.197	0.191
Gender									
Boys	12 (17.4)	2 (15.4)	5 (19.2)	18 (20.0)	24 (21.8)	13 (18.8)	1 (14.3)	1 (11.1)	13 (18.1)
Girls	57 (82.6)	11 (84.6)	21 (80.8)	72 (80.0)	86 (78.2)	56 (81.2)	6 (85.7)	8 (88.9)	59 (81.6)
*p*- value	0.011	0.292	0.280	0.013	0.014	0.028	0.410	0.247	0.013
Remoteness									
Cities	48 (69.6)	10 (76.9)	15 (57.7)	59 (65.5)	71 (64.5)	47 (68.1)	3 (42.9)	7 (77.8)	49 (68.0)
Regional/Remote	21 (30.4)	3 (23.1)	11 (42.3)	31 (34.4)	39 (35.5)	22 (31.9)	4 (57.1)	2 (22.2)	23 (31.9)
*p*- value	0.050	0.213	0.731	0.168	0.161	0.101	0.323	0.281	0.092
Household income									
Low	25 (36.2)	3 (23.1)	13 (50.0)	20 (22.2)	31 (28.2)	19 (27.5)	3 (42.9)	2 (22.2)	19 (26.4)
Medium	31 (44.9)	7 (53.8)	9 (34.6)	45 (50.0)	52 (47.3)	35 (50.7)	3 (42.9)	6 (66.7)	36 (50.0)
High	13 (18.9)	3 (23.1)	4 (15.4)	25 (27.8)	27 (24.5)	15 (21.8)	1 (14.2)	1 (11.1)	17 (23.6)
*p*- value	0.234	0.878	0.040	0.046	0.554	0.905	0.697	0.524	0.773
Parents’ education									
Bachelor	15 (21.7)	3 (23.0)	6 (23.0)	26 928.9)	28 (25.4)	17 (24.7)	2 (28.6)	2 (22.2)	19 (26.4)
Diploma	30 (43.5)	5 (38.5)	10 (38.5)	33 (36.7)	42 (38.2)	29 (42.0)	3 (42.8)	4 (44.5)	30 (41.7)
Year 10/11	24 (34.8)	5 (38.5)	10 (38.5)	31 (34.4)	40 (36.4)	23 (33.3)	2 (28.6)	3 (33.3)	23 (31.9)
*p*- value	0.353	0.914	0.821	0.826	0.604	0.715	0.952	0.916	0.787
Parents’ employment									
Employed	37 (53.6)	10 (76.9)	14 (53.8)	64 (71.1)	74 (67.3)	48 (69.6)	5 (71.4)	6 (66.7)	50 (69.4)
Unemployed	32 (46.4)	3 (23.1)	12 (46.2)	26 (28.9)	36 (32.7)	21 (30.4)	2 (28.6)	3 (33.3)	22 (30.6)
*p*- value	<0.001	0.583	0.047	0.790	0.247	0.873	0.944	0.810	0.846
Family type									
Original	34 (49.3)	4 (30.8)	8 (30.8)	49 (54.5)	55 (50.0)	32 (46.4)	3 (42.8)	4 (44.5)	35 (48.6)
Other	35 (50.7)	9 (69.2)	18 (69.2)	41 (45.5)	55 (50.0)	37 (53.6)	4 (57.1)	5 (55.5)	37 (51.4)
*p*- value	0.678	0.125	0.023	0.365	0.671	0.297	0.652	0.677	0.562
Family functioning									
Very good/Good	49 (71.0)	9 (69.2)	20 (76.9)	65 (72.2)	79 (71.8)	43 (62.3)	7 (100.0)	7 (77.8)	46 (63.9)
Fair/Poor	20 (28.9)	4 (30.8)	6 (23.1)	25 (27.8)	31 (28.2)	26 (37.7)	0 (0.0)	2 (22.2)	26 (36.1)
*p*- value	0.697	0.775	0.592	0.901	0.749	0.012	0.097	0.721	0.028
IRSAD quintile									
Lowest (Most disadvantaged)	18 (26.1)	4 (30.8)	9 (34.6)	18 (20.0)	24 (21.8)	12 (17.4)	2 (28.5)	1 (11.1)	12 (16.7)
Second	7 (10.2)	1 (7.7)	2 (7.7)	10 (11.1)	12 (10.9)	12 (17.4)	1 (14.3)	1 (11.1)	12 (16.7)
Third	17 (24.6)	2 (15.4)	6 (23.1)	22 (24.4)	29 (26.4)	16 (23.2)	1 (14.3)	4 (44.5)	18 (25.0)
Fourth	11 (15.9)	2 (15.4)	4 (15.4)	16 (17.8)	19 (17.3)	10 (14.5)	1 (14.3)	1 (11.1)	10 (13.9)
Highest (Most advantaged)	16 (23.2)	4 (30.7)	5 (19.2)	24 (26.7)	26 (23.6)	19 (27.5)	2 (28.5)	2 (22.2)	20 (27.8)
*p*- value	0.402	0.734	0.471	0.131	0.056	0.428	0.967	0.572	0.263
Substance Use by the child									
No	10 (14.5)	2 (15.4)	5 (19.2)	14 (15.6)	17 (15.5)	7 (10.1)	0 (0.0)	2 (22.2)	9 (12.5)
Yes	59 (85.5)	11 (84.6)	21 (80.8)	76 (84.4)	93 (84.5)	62 (89.9)	7 (100.0)	7 (77.8)	63 (87.5)
*p*- value	0.091	0.615	0.827	0.070	0.018	0.004	0.166	0.916	0.021
Any Mental disorder									
No	16 (23.2)	3 (23.1)	11 (42.3)	32 (35.6)	39 (35.5)	9 (13.0)	1 (14.3)	2 (22.2)	11 (15.3)
Yes	53 (76.8)	10 (76.9)	15 (57.7)	58 (64.4)	71 (64.5)	60 (87.0)	6 (85.7)	7 (77.8)	61 (84.7)
*p*- value	<0.001	0.170	0.889	0.119	0.042	<0.001	0.141	0.237	<0.001
Suicidality group									
Ideation without plan or attempt	16 (23.2)	1 (7.7)	7 (26.9)	27 (30.0)	32 (29.1)	14 (20.3)	1 (14.3)	2 (22.2)	14 (19.4)
Ideation with planned and/or attempted	33 (76.8)	12 (92.3)	19 (73.1)	63 (70.0)	78 (70.9)	55 (79.7)	6 (85.7)	7 (77.8)	58 (80.6)
*p*- value	0.379	0.106	0.986	0.312	0.353	0.112	0.446	0.751	0.063

Data are shown as n (%)

P-value of association with different mental health services

Table 5 highlights the mental health service use of among suicidal adolescents aged 13–17 years. Age of adolescents only had a significant impact on their use of telephone services in parent data. While gender was significantly associated with health service and online service use as reported in child data, and with any service as reported by parent data. Child data also shows that adolescents from unemployed parents’ were 5.88 times (95% CI: 2.07–16.68) more likely to use health services compared to those from working parents’. Substance use among suicidal adolescents was only found to be significantly associated with health service use (OR 4.55, 95% CI: 1.34–15.44), as reported in child data. Both child data and parents reported that the most used service for suicidal adolescents with a mental disorder was health service compared to those who do not have any mental disorder. While, only according to the parents’, any service was 2.38 times (95% CI: 1.09–5.17) more likely to be accessed by adolescents who reported suicidal ideation with planned and/or attempted compared to those who reported only ideation.

**Table 5:** Factors associated with mental health service uses in sub-sample II (binary regression).

Sociodemographic factors		Child data					Parent data			
		Health Service	School Service	Telephone Service	Online Service	Any Service	Health Service	School Service	Telephone Service	Any Service
		OR (95% CI)	OR (95% CI)	OR (95% CI)	OR (95% CI)	OR (95% CI)	OR (95% CI)	OR (95% CI)	OR (95% CI)	OR (95% CI)
Age (Ref. 13 to ≤15)	>15 to 17	0.73 (0.30, 1.79)	0.34 (0.09, 1.32)	1.21 (0.41, 3.55)	1.78 (0.79, 4.00)	1.72 (0.77, 3.87)	0.96 (0.41, 2.24)	0.03 (0.00, 1.02)	11.47***(1.29, 101.57)	0.89 (0.38, 2.08)
Gender (Ref. Boys)	Girls	2.75***(1.04, 7.29)	2.24 (0.47, 10.62)	2.02 (0.46, 8.86)	2.39***(1.11, 5.13)	2.21 (0.98, 4.94)	2.18 (0.88, 5.36)	3.18 (0.08, 113.43)	3.13 (0.20, 47.75)	2.41***(1.01, 5.74)
Remoteness (Ref. Cities)	Regional/Remote	0.71 (0.32, 1.59)	0.58 (0.12, 2.73)	1.76 (0.61, 5.10)	0.88 (0.41, 1.85)	1.17 (0.51, 2.65)	0.74 (0.31, 1.72)	2.89 (0.50, 16.71)	0.61 (0.13, 2.78)	0.81 (0.36, 1.86)
Household income (Ref. Low)	Medium	0.46 (0.15, 1.41)	2.07 (0.31, 13.55)	0.34 (0.09, 1.30)	1.60 (0.64, 3.98)	0.82 (0.29, 2.36)	1.01 (0.35, 2.86)	0.44 (0.00, 85.36)	1.75 (0.19, 15.47)	1.00 (0.35, 2.82)
	High	0.40 (0.10, 1.61)	2.66 (0.25, 28.25)	0.29 (0.04, 1.74)	2.62 (0.71, 9.60)	1.29 (0.29, 5.60)	0.51 (0.12, 2.13)	0.57 (0.00, 43.74)	0.24 (0.01, 5.02)	0.78 (0.19, 3.18)
Parents’ education (Ref. Bachelor)	Diploma	0.81 (0.27, 2.39)	0.93 (0.15, 5.73)	0.91 (0.17, 4.74)	1.21 (0.42, 3.45)	1.32 (0.44, 3.98)	0.36 (0.10, 1.23)	0.16 (0.01, 1.89)	0.72 (0.10, 5.03)	0.35 (0.10, 1.16)
	Year 10/11	0.63 (0.10, 1.61)	0.90 (0.14, 5.57)	0.91 (0.19, 4.40)	2.26 (0.71, 7.11)	2.86 (0.83, 9.84)	0.45 (0.10, 1.98)	0.81 (0.04, 15.36)	1.89 (0.39, 9.01)	0.40 (0.09, 1.69)
Parents’ employment (Ref. Employed)	Unemployed	5.88**(2.07, 16.68)	0.75 (0.09, 6.11)	1.58 (0.48, 5.18)	1.16 (0.47, 2.84)	1.90 (0.71, 5.10)	1.14 (0.43, 3.06)	1.56 (0.24, 9.87)	1.08 (0.18, 6.31)	1.17 (0.44, 3.10)
Family type (Ref. Original)	Other	1.12 (0.46, 2.69)	4.11 (0.95, 17.74)	3.59 (1.29, 10.02)	0.85 (0.40, 1.79)	1.47 (0.64, 3.38)	2.04 (0.81, 5.12)	0.69 (0.06, 7.29)	1.30 (0.37, 4.58)	1.73 (0.73, 4.09)
Family functioning (Ref. Very good/Good)	Fair/Poor	0.80 (0.33, 1.88)	1.07 (0.26, 4.36)	0.60 (0.17, 2.15)	0.94 (0.42, 2.09)	0.80 (0.35, 1.82)	1.36 (0.62, 2.97)	-	0.41 (0.07, 2.41)	1.18 (0.54, 2.56)
IRSAD quintiles (Ref. Lowest)	Second	0.27* (0.07, 1.02)	0.21 (0.01, 3.02)	0.32 (0.05, 2.02)	0.73 (0.22, 2.38)	0.55 (0.13, 2.33)	1.27 (0.28, 5.77)	2.43 (0.01, 442.14)	0.91 (0.03, 23.49)	1.30 (0.31, 5.47)
	Third	1.41 (0.40, 4.96)	0.49 (0.06, 3.99)	1.71 (0.28, 10.29)	1.13 (0.38, 3.34)	2.36 (0.70, 7.99)	1.06 (0.28, 4.07)	4.38 (0.03, 620.39)	4.37 (0.36, 51.67)	1.48 (0.39, 5.52)
	Fourth	2.41 (0.62, 9.37)	0.62 (0.07, 5.14)	1.68 (0.41, 6.91)	1.44 (0.52, 3.94)	2.47 (0.71, 8.52)	1.68 (0.40, 7.04)	0.69 (0.02, 19.64)	2.04 (0.09, 43.91)	1.38 (0.33, 5.69)
	Highest	2.03 (0.51, 8.03)	0.83 (0.09, 7.46)	2.47 (0.55, 10.92)	2.05 (0.61, 6.87)	3.15 (0.86, 11.51)	2.54 (0.60, 10.77)	4.17 (0.14, 121.74)	1.75 (0.15, 20.01)	2.03 (0.51, 8.03)
Substance Use (Ref. No)	Yes	4.55**(1.34, 15.44)	2.79 (0.28, 27.88)	1.03 (0.28, 3.77)	1.76 (0.67, 4.66)	2.18 (0.80, 5.92)	3.11 (0.74, 12.94)	-	0.67 (0.13, 3.45)	2.61 (0.71, 9.50)
Mental disorder (Ref. No.)	Yes	4.88*(2.17, 10.98)	4.32 (0.74, 24.93)	0.83 (0.28, 2.43)	1.60 (0.78, 3.28)	1.81 (0.80, 4.08)	12.01**(3.69, 39.05)	10.79**(1.81, 64.29)	3.58 (0.33, 38.05)	9.67*(3.31, 28.24)
Ideators (Ref. Ideation without plan or attempt)	Ideation with planned and/or attempted	1.39 (0.49, 3.90)	5.29 (0.35, 79.67)	1.05 (0.34, 3.17)	0.72 (0.32, 1.62)	0.57 (0.22, 1.48)	1.98 (0.90, 4.32)	1.91 (0.06, 60.56)	1.81 (0.52, 6.33)	2.38***(1.09, 5.17)

OR = odds ratio; CI = confidence interval

Level of Significance Considered: P<0.05***, P<0.01**, P<0.001*

Survey weight adjusted

## Discussion

Building on previous research on mental health service use in adolescents, the findings of this study suggest the differences in the factors influencing the service use among adolescents aged 13–17 with a mental disorder and/or suicidality.

Mental disorders and/or suicidality are frequent in adolescents aged 13–17; however, mental health services were less commonly accessed by these adolescents. The results suggest that service use in adolescents with a mental disorder was relatively low (about 27–30%) in comparison to previous study findings, which varies between 33–40% [26, 34–36]. In case of suicidal adolescents, a significant proportion (34.5% in child data and 57.1% in parent data) did not use any services, which is similar to the previous population-based studies in the US and Canada[13, 37]. This ongoing disparity may be explained by the shortage of mental health resources and the lack of training of health professionals in the detection of mental health problems among adolescents [4, 13]. Though, it is important to note that the quality of the mental health care provided was not measured in this study.

The findings of the study shows that several factors were significantly associated with the service use in adolescents aged 13–17 with mental disorders and/or suicidality. Older age group (>15 to 17 years) tended to use more mental health services than to younger group (13 to ≤15 years). One explanation for this may be that older age-group is more likely to be in situations (e.g. access to the Internet and telephone) where they can use mental health services easily compared to younger group [4]. Consistent with previous research [28, 38, 39], the present study has also shown that girls than boys either with mental disorder or suicidality were more likely to use mental health services. This is may be due to the fact that girls are experiencing more mental health problems than boys in the adolescence [4]. Amongst other factors, the fact that fewer boys were using mental health services could be attributed to their lower perceived need for mental health support due to mental health problems, their preference for self-management and their lack of knowledge about where to get services, and more negative attitudes towards health service providers [39–41]. The results also show that adolescents with mental disorders and suicidality living in families with unemployed parents, low-medium income, or with other families (i.e. combination of sole, step and blended parent) accessed more mental health services than those in families that were least disadvantaged. Previous studies claimed that the service use is higher among disadvantaged families because mental health problems are more prevalent among adolescents in these families [15, 26]. Interestingly, the findings also suggest that substance use by the adolescents is significantly associated with service use in both subgroups, which is not similar with the previous research [42] and shows the increasing importance of service use among adolescents with the habit of substance use. Also, confirming the previous research [43], the present study shows that adolescents with multiple mental disorders more likely to access health services, school services or any services compared to those with single mental disorder. This is may be because individuals with multiple mental disorders undergo more adverse social consequences such as stigma, stress, social withdrawal, family conflict, and financial problems, and as a result seeking professional help for mental health problems [43, 44]. Furthermore, among suicidal adolescents, suicidal ideation with planned and/or attempted group accessed more mental health services compared to ideators group only. However, these differences were not statistically significant, which most likely results due to small sample size. Overall, results indicated that the increased service use by adolescents with mental disorders and/or suicidality was noticeable for health services and online services, which is corroborated and expanded on those from previous studies in Australia [26, 45]. Further, data revealed that adolescents in both subpopulations did not use school services and telephone services as they were expected, which may results from an uptake of online service among adolescents [26]. Further research is needed supporting the efficacy of school and telephone services in adolescents aged 13–17 with mental disorders and/or suicidality.

This study has a few limitations that deserve to be mentioned. First, information on suicidality was from self-reported child data and not based on validated screening/assessment tools; hence stigma may have resulted in underreporting. Second, some determinants such as duration, waiting times, past experiences with accessed services were not included in the analysis, which can be serious barriers to mental health service uses [38]. The study is also limited by the fact that it did not examine whether the service use improved the mental health problems in adolescents, their performance in schools and social functioning [3]. Lastly, the reliability of recall as the analysis was based on a cross-sectional data which limits causal inferences. However, this is the most plausible method, and has been validated in earlier studies and accepted by experts in the field [46]. However, findings of the study provided a better understanding of the several factors that have an impact on mental health service use in adolescents with mental disorder and suicidality using nationally representative data.

## Conclusion

For many adolescents experiencing mental disorders and/or suicidality, mental health service uses remain low; which may be fueled by the lack of understanding of the factors that affecting mental health services in adolescents aged 13–17 years. The results indicated that some factors must be more clearly understood to facilitate increased service use by adolescents with mental disorder and/or suicidality. For instance, in adolescents with mental disorder and suicidality, there is inequitable access to services among subgroups such boys and adolescents from disadvantaged families. Innovative initiatives are necessary to reach this community. Additional research should address the specific barriers that may limit the use of mental health services, school and telephone counseling services in particular.
